# Design of Magnetic Nanoplatforms for Cancer Theranostics

**DOI:** 10.3390/bios12010038

**Published:** 2022-01-12

**Authors:** Wangbo Jiao, Tingbin Zhang, Mingli Peng, Jiabao Yi, Yuan He, Haiming Fan

**Affiliations:** 1 Key Laboratory of Synthetic and Natural Functional Molecule Chemistry of the Ministry of Education, College of Chemistry and Materials Science, Northwest University, Xi’an 710069, China; jwbzzh@stumail.nwu.edu.cn (W.J.); zhangtb@nwu.edu.cn (T.Z.); mlpeng@nwu.edu.cn (M.P.); 2 Global Innovative Centre for Advanced Nanomaterials, School of Engineering, The University of Newcastle, Newcastle, NSW 2308, Australia; jiabao.yi@newcastle.edu.au

**Keywords:** iron oxide nanoparticles, magnetotheranostics, cancer

## Abstract

Cancer is the top cause of death globally. Developing smart nanomedicines that are capable of diagnosis and therapy (theranostics) in one–nanoparticle systems are highly desirable for improving cancer treatment outcomes. The magnetic nanoplatforms are the ideal system for cancer theranostics, because of their diverse physiochemical properties and biological effects. In particular, a biocompatible iron oxide nanoparticle based magnetic nanoplatform can exhibit multiple magnetic–responsive behaviors under an external magnetic field and realize the integration of diagnosis (magnetic resonance imaging, ultrasonic imaging, photoacoustic imaging, etc.) and therapy (magnetic hyperthermia, photothermal therapy, controlled drug delivery and release, etc.) in vivo. Furthermore, due to considerable variation among tumors and individual patients, it is a requirement to design iron oxide nanoplatforms by the coordination of diverse functionalities for efficient and individualized theranostics. In this article, we will present an up–to–date overview on iron oxide nanoplatforms, including both iron oxide nanomaterials and those that can respond to an externally applied magnetic field, with an emphasis on their applications in cancer theranostics.

## 1. Introduction

Due to the huge differences between individual patients, a tough question exists in the field of tumor diagnosis and therapy: when and where to apply what kind of treatment for a particular patient? Image–guided therapy, known as theranostics, provides a new solution for this problem. The integration of diagnosis and therapy means that treatment can be carried out under the guidance of images and monitored in real time to achieve precise and personalized medical treatment. Due to their diversified functions, nanomaterials provide a great opportunity for the integration of efficient diagnosis and therapy into a single nanoplatform [1]. There are several requirements for an ideal theranostics nanoplatform. Firstly, the nanoplatform should possess good diagnostic and/or therapeutic capabilities. Secondly, this nanoplatform must be able accumulate at the target area. Thirdly, the biocompatibility of this nanoplatform must be acceptable. Finally, it should have the ability to be integrated with other diagnostic and/or therapeutic technologies for multi–modality theranostics. Magnetotheranostics is a kind of advanced medical technology that utilizes the interaction between magnetic nanoplatforms and magnetic fields to realize the integration of therapy and diagnosis on a single nanoparticle. The magnetic nanoplatforms are known to have excellent biocompatibility [2,3], diversified diagnostic and therapeutic capabilities [4], as well as active/passive targeting capabilities [5,6], while the magnetic field is well recognized for no attenuation [7] and little damage to the tissue [8]. As a result, magnetotheranostics has received a great deal of attention in cancer research recently.

**Figure 1:**
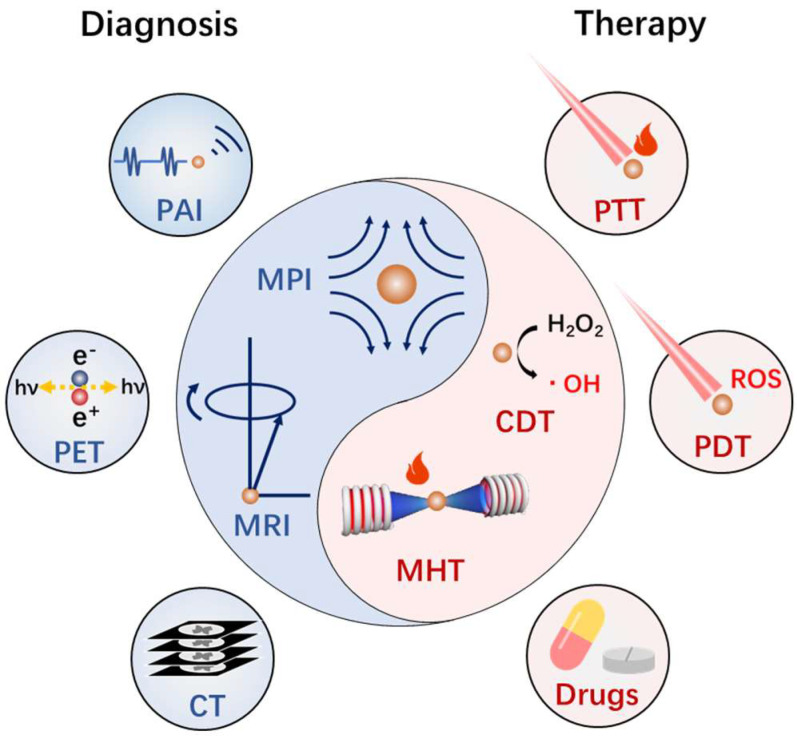
Diverse diagnosis and treatment technologies based on functionalized MIONs.

As the core of magnetotheranostics nanoplatforms, magnetic iron oxide nanoparticles (MIONs) themselves have the ability to achieve diagnosis and therapy. The classic example of MIONs for diagnosis is magnetic resonance imaging (MRI) contrast agent [9]. Some iron oxide–based MRI contrast agents have been clinically approved, such as Ferrixan and Ferumoxide [10]. MIONs can achieve T1–weighted or T2–weighted MRI enhancement by affecting the T1 or T2 relaxation rate of surrounding protons. Although MIONs are generally considered to have only single–modal imaging capabilities, the development of advanced imaging technologies based on MIONs such as magnetic particle imaging (MPI) and magnetomotive optical coherence tomography (MMOCT) has also greatly enriched the imaging prospect of MIONs [11,12]. On the other hand, therapy technologies based on MIONs are gradually being developed. Magnetic hyperthermia (MHT) is a therapy method through the ability of MIONs to convert the energy of alternating magnetic field (AMF) into heat. In Europe, Nanotherm^®^, which is used as a nano agent of magnetic hyperthermia for brain gliomas, has been approved for clinical use [13]. MIONs also have the ability to kill cancer cells by producing reactive oxygen species (ROS) through the Fenton reaction catalyzed by Fe^2+^, which is known as chemodynamic therapy (CDT) [14]. Additionally, MIONs can integrate with other materials for multi–modality therapy or diagnostics (Figure 1). For example, gold–magnetic composite nanomaterials are used as optical–magnetic hybrid nano–platforms for the integration of PDT, PTT, and MRI, and even CT and PET [15,16,17]. Appropriate surface modification can bring better biocompatibility [18], blood circulation time [19], active targeting [20], and even additional therapeutic and diagnostic functions [21] for MIONs. When combined with functional molecules, MION could satisfy more diverse demands. Fluorescent molecules can give MIONs the ability of fluorescence imaging [22]. Another example is that MIONs can carry and deliver drugs to the tumor area through some tumor–targeting methods [23].

In this review, we summarized the progress in magnetotheranostics nanoplatforms for cancers in recent years. First, we reviewed the synthetic techniques of MIONs. Then, we introduced the design of therapy and diagnosis technologies (e.g., MRI, MHT, CDT, and others) based on MIONs. The next part of this review will focus on the design strategy of magnetotheranostics nanoplatforms combined with other therapy or diagnostic platforms, including phototheranostics, computed tomography (CT), positron emission computed tomography/single photon emission computed tomography (PET/SPECT), fluorescence imaging (FI), and drug delivery. The summary of the design strategy from magnetic function unit to external modification will help to deepen the understanding on their therapy and diagnostic capabilities. We hope this can provide some new ideas for the future design of new magnetotheranostics nanoplatforms.

## 2. Controlled Synthesis of Magnetic Nanoplatforms

The magnetic function unit of magnetotheranostics mainly refers to MIONs. MIONs can be synthesized by physical, biological, and chemical means. Physical methods include ball milling, vapor deposition, photolithography and other technologies, but the properties of MIONs synthesized by physical methods are difficult to control [24]. The biosynthesis of MIONs has some advantages, such as better environmental friendliness and product biocompatibility, but it also faces the problems of low crystallinity and difficulty in controlling the size and morphology [25]. Chemical synthesis of MIONs is the most commonly used method. Starting from the initial co–precipitation method [26,27], researchers have successively developed thermal decomposition methods [28], hydrothermal methods [29], solvothermal methods [30], sol–gel methods [31], Micelle methods [32], and other methods to construct MIONs. 

**Figure 2:**
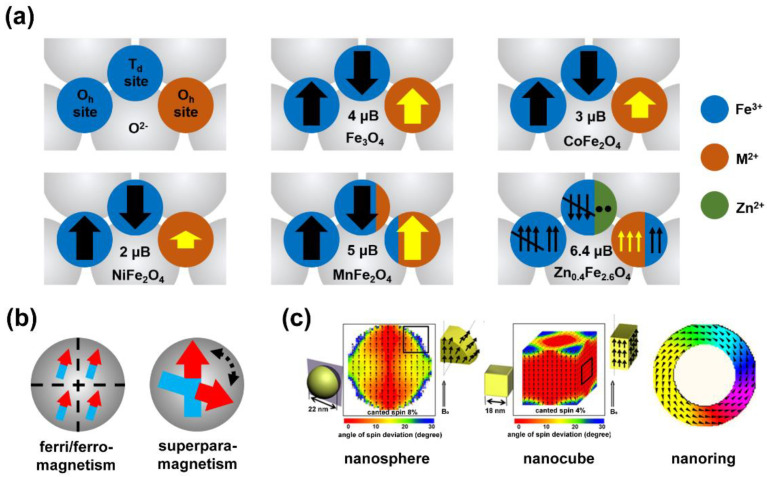
(**a**) Structure schematic of spinel structure and magnetic spin of MFe_2_O_4_. (**b**) Magnetic domain of ferrimagnetism/ferromagnetism (**left**) and superparamagnetism (**right**). (**c**) Magnetic spin states simulated using OOMMF program for nanosphere and nanocube [37] and vortex domain structure of nanoring simulated by the LLG Micromagnetics Simulator^TM^ package [38]. Reprint permission from [37,38]. Copyright 2012 American Chemical Society and 2012 American Institute of Physics.

MIONs are a type of iron–based metal oxide nanoparticles with a spinel structure, whose composition can be expressed as MFe_2_O_4_, and M represents divalent metal ions, including Mn^2+^, Fe^2+^, Co^2+^, Ni^2+^, Zn^2+^, etc. (Figure 2a). In the most common Fe_3_O_4_ materials, M = Fe^2+^ and the Fe^2+^ occupies the octahedral (O_h_) sites of the spinel structure, forming an inverse spinel structure. The antiferromagnetic coupling between Fe^3+^ makes the overall magnetic spin behave as 4 μB of Fe^2+^. The conditions of CoFe_2_O_4_ and NiFe_2_O4 are similar to those of Fe_3_O_4_ materials, and the total magnetic spins are 3 μB of Co^2+^ and 2 μB of Ni^2+^ respectively. When M = Mn^2+^, Mn^2+^ mainly occupies octahedral sites and partly occupies the tetrahedral (T_d_) sites, forming a mixed spinel structure. However, since the magnetic spins of Mn^2+^ and Fe^3+^ are both 5 μB, they always show a total magnetic spin of 5 μB in the end [33]. In ZnFe_2_O_4_, Zn^2+^ occupies a tetrahedral position to form a normal spinel structure. The magnetic spin of Zn^2+^ is 0 μB, and the magnetic spins of two Fe^3+^ cancel each other out, showing 0 μB overall, theoretically. Interestingly, in Zn_0.4_Fe_2.6_O_4_, the antiferromagnetic coupling between Fe^3+^ is broken by the configuration reversal caused by the partial doping of Zn^2+^, and shows a higher remanent magnetic spin [34]. The composition control of MIONs can be easily achieved by adjusting the ratio of metal precursors. In the thermal decomposition method, this usually depends on the feeding amount of different metal–organic complexes. For example, Sun et al. synthesized MnFe_2_O_4_ and CoFe_2_O_4_ nanoparticles by thermal decomposition of manganese acetylacetonate, cobalt acetylacetonate with iron(III) acetylacetonate [35]. Long–chain fatty acid complexes are another type of common organic precursors. Zhang et al. used iron(III) erucate and manganese oleate, cobalt oleate to synthesize MnFe_2_O_4_ and CoFe_2_O_4_ nanoparticles [36]. In other synthesis methods, MFe_2_O_4_ is usually synthesized by directly adding different metal ions. Co–precipitation and solvothermal methods can be used to generate MFe_2_O_4_ by adding Mn^2+^, Co^2+^, Ni^2+^, or Zn^2+^ [26,29].

In addition to the influence of the crystal structure itself, the magnetic properties of MIONs are greatly affected by their size. Excluding the influence of other factors, the smaller the MIONs, the lower the saturation magnetization (Ms) [39]. When the size is smaller than the critical size, the magnetic anisotropy of MIONs is not enough to resist the effects of thermal disturbance, resulting in the loss of its own remanence and hysteresis, but still maintaining a high initial magnetic susceptibility. This phenomenon is called superparamagnetic (Figure 2b). Due to its zero remanence, superparamagnetic iron oxide nanoparticles (SPIOs or SPIONs) have excellent colloidal dispersion and better stability than ferromagnetic or ferrimagnetic nanoparticles, and they have been approved for clinical use [10]. The size of MIONs can be controlled by the reaction temperature and the amount of surfactant. Park et al. used several solvents with different boiling points to control the reaction temperature in thermal decomposition of iron oleate, including 1–hexadecene (b.p. 274 °C), octyl ether (b.p. 287 °C), 1–octadecene (b.p. 317 °C), 1–eicosene (b.p. 330 °C), and trioctylamine (b.p. 365 °C), and successfully synthesized Fe_3_O_4_ nanoparticles of 5–22 nm sizes [28]. Xu et al. [40] used iron acetylacetonate to synthesize 7–10 nm Fe_3_O_4_ nanoparticles by adjusting the ratios of oleylamine and benzyl ether in the high temperature thermal decomposition process.

The morphology will also affect the magnetic properties of MIONs (Figure 2c). Compared with spherical MIONs, cubic MIONs have a higher Ms due to the less distributed spin disorder layer on the surface [37,41]. The ring–shaped MIONs possess a unique vortex magnetic domain [38], enabling it to have zero remanence and zero hysteresis while maintaining ferrimagnetism [42]. To synthesize MIONs with a specified morphology, which means to achieve anisotropic growth of MIONs, it is necessary to provide a near thermodynamically stable environment during crystal growth so that the interface energy of different crystal faces dominates the process. Therefore, the usage of thermal decomposition method or hydrothermal method are more appropriate choices. In the work of Zhou et al., sodium oleate was used to control the growth of different crystal faces during the thermal decomposition of iron oleate to obtain cubes, concaves, multibranch shaped MIONs [43]. An example of hydrothermal control of the morphology of MIONs is the synthesis of α–Fe_2_O_3_ nanorings and nanotubes by phosphate and sulfate dianion–assisted hydrothermal method, and controlled synthesis of Fe_3_O_4_ and γ–Fe_2_O_3_ nanorings/tubes by thermal reduction and thermal oxidation [44].

The naked MIONs may not be suitable for direct biological application. During or after the preparation of MIONs, they need to be further modified to endow them with better stability for biological applications. Polymers are a kind of widely used coatings, including synthetic polyethylene glycol (PEG) [18,45], polyethyleneimine (PEI) [46], polyacrylic acid (PAA) [47], polyvinylpyrrolidone (PVP) [48] and natural dextran [19], chitosan [49], alginate [50], and so on. For example, modification of PEG not only enhances the colloidal dispersion and stability of MIONs and improves their biocompatibility, but also prolongs the average time for MIONs to be recognized and swallowed by macrophages in the liver and spleen, which will extend the blood circulation time of MIONs [18]. Dextran is shown to have a similar effect [19]. Silane is also a common coating of MIONs. Amino silane is used for surface modification of the magnetic hyperthermia agent NanoTherm^®^ [51]. The modification with antibodies [20], targeting peptides [52] or other targeting molecules [53] can provide MIONs with active targeting capability. Meanwhile, surface modifications may also affect the therapeutic and diagnostic functions of MIONs. The thickness of the surface coating has been proven to affect the T2 relaxation performance of MIONs [54] and the performance of magnetic hyperthermia [55]. In the study of Zeng et al. [56], the difference in anchor groups can also affect its Ms and further affect its T2 relaxation properties. Connecting graphene oxide on the surface of MIONs can improve its magnetothermal effect in the form of dielectric loss [57]. Surface ligands are often used to control the spatial position between various groups [58]. PEG coatings of different molecular weights have been used to precisely adjust the distance between natural enzymes and MIONs nanozymes [59]. Self–assembled monolayers (SAMs) can also control the spatial distribution of surface functional groups at the molecular level [60]. Modification of detection molecules on the surface allows MIONs to be widely used in the diagnosis of various biomolecules in vitro, including circulating tumor cells (CTC), alpha–fetoprotein, ctDNA, and other markers. The target cell/molecules can be captured by MIONs, magnetically separated and then detected by polymerase chain reaction (PCR), enzyme–linked immunosorbent assay (ELISA), or more sensitive atomic force microscope (AFM)–based technology [61,62].

## 3. Basis of Magnetic Nanomaterials Mediated Diagnosis and Therapy of Cancer

The magnetic properties of MIONs magnetic core can affect the relaxation process of protons, making it useful for MRI contrast agents. MIONs can absorb the energy of the magnetic field to generate in situ heating under the alternating magnetic field, and then can realize the magnetic hyperthermia of the tumor. The Fenton reaction by Fe^2+^ enables the generation of ROS to mediate tumor chemodynamic therapy. These physicochemical properties can be applied for cancer diagnosis and therapy. Together with the low toxicity [2] and clear degradation metabolism [3], MIONs have received increasing attention for theranostics.

### 3.1. Biosafety of Magnetic Nanoplatforms

MION formulations are generally considered to have excellent biological safety. Naked MIONs have strong antigenicity and are prone to cause allergic reactions. Surface modification such as dextran can significantly avoid side effects. There have been a large number of studies to evaluate the possible side effects of MIONs for clinical use so far. In the cell viability studies, most MIONs reported only showed cytotoxicity at particularly high concentrations [63,64,65]. For example, the ability of nerve cells to lengthen neurons was reduced in a dose–dependent manner by Fe. In this example, anionic magnetic nanoparticles are applied [66]. Animal experiments showed that the LD50 of MIONs is indeed affected by its surface modification. The LD50 of naked MIONs is 300~600 mg/kg. When MIONs were coated with dextran, the LD50 are increased to 2000~6000 mg/kg [67]. When used to treat lymph node metastases from thoracic squamous cell carcinoma of the esophagus, MIONs (ferucarbotran in this case) exhibited negligible side effects [68]. The application of dextran–coated MIONs in the diagnosis of carotid inflammatory plaques also showed no obvious side effects [69]. However, some adverse reactions in the clinical study of ferumoxtrans–10 were observed, and even one case died after the injection of undiluted MIONs [70]. The report pointed out that the safety of MIONs is highly dependent on the dose used. The death may be caused by the rapid formation of aggregated particles in undiluted MIONs, which then accumulate in the kidney and liver through phagocytes to cause acute toxicity [71].

### 3.2. Magnetic Resonance Imaging

Due to its high soft tissue contrast, high temporal and spatial resolution, and no ionizing radiation, MRI is widely used for imaging of soft tissues such as brain, heart, muscle, and tumor [72]. MRI signals are derived from nuclear magnetic resonance (NMR) signals from water protons in human tissues. Depending on the received proton longitudinal relaxation (T1) or transverse relaxation (T2) signals, MRI imaging methods are divided into two types: T1 weighting and T2 weighting. These relaxation signals can be affected by the magnetic properties of MIONs, thereby enhancing their signal strength and improving the contrast between diseased tissues and normal tissues. The contrast agents for these two imaging methods are thus called T1 contrast agents and T2 contrast agents, respectively, and their ability to enhance the corresponding relaxation rate is characterized by r1 and r2 values. The superparamagnetism of SPIONs makes them capable of disturbing the magnetic uniformity near itself under the high main magnetic field conditions of MRI, which can accelerate the lateral relaxation of surrounding protons, reduce the signal intensity in T2–weighted magnetic resonance images, and achieve negative image enhancement. Although some SPIONs were approved for clinical use as pure T2 contrast agents, they have been gradually withdrawn from clinical application in recent years, mainly due to shortcomings of poor imaging specificity (such as confusion with bleeding and calcification) [73]. Nevertheless, the T2 contrast enhancement of MIONs has been widely used in recent years for image tracking and therapy guidance of the spatiotemporal position of MIONs in vivo, such as the use of T2–weighted MRI to track cells marked by MIONs [74,75].

T1–weighted MRI can avoid the shortcomings of T2–weighted MRI, so it has better clinical usage. During the relaxation process, the protons can transfer energy with the T1 contrast agent to shorten its T1 relaxation time, especially for Gd^3+^, Fe^3+^, Mn^2+^, and other ions containing a large number of unpaired valence electrons. In comparison to the clinically used Gd–based contrast agents with biological safety problems [76,77], MIONs are well recognized for better biocompatibility, and Fe^3+^ grants them potential T1 imaging capabilities. However, large–sized MIONs have high Ms and T2 enhanced imaging performance. The high r2/r1 ratio limits their application in T1 contrast imaging. The emergence of ultrasmall SPIONs provides an opportunity to solve this problem. When the size of MIONs is reduced, their Ms decreases sharply. This reduces the r2 value, meanwhile the increased surface area increases its r1 value, leading to declined r2/r1 ratio to the range that allows T1 imaging. With the progress in new technologies for the large–scale synthesis of ultrasmall SPIONs [36,78], the clinical application of ultrasmall SPIONs as T1 contrast agents has also rapidly developed. Wei et al. [79] reported the synthesis of a zwitterion (ZES) coated ultrasmall SPIONs with a magnetic core diameter of about 3 nm, a hydrophilic shell thickness of about 1 nm, a low r2/r1 value and a long internal circulation time, for high resolution T1 MRI imaging of vessels with a spatial resolution of about 0.2 mm. Miao et al. [80] studied the effect of different doping of the core–shell structure on the T1 imaging performance of ultrasmall SPIONs. The optimized 3.8 nm Zn_x_Fe_3−x_O4@Zn_x_Mn_y_Fe_3−x−y_O_4_ core–shell ultrasmall SPIONs has an r1 relaxation rate of 20.22 mM^−1^s^−1^, which is 5.2–fold and 6.5–fold larger than that of the undoped ultrasmall SPIONs and the clinically used Gd–DTPA. Such nanoagent was then shown to be able to detect micro metastases in the lungs of mice. In order to extend the circulation time in vivo, some MIONs are designed to form larger aggregates in the form of clusters before entering the target area, showing T2 enhanced performance. After reaching the target area, it disintegrates into ultrasmall SPIONs responsively, realizing the conversion of T2 to T1 contrast enhancement [81]. This mode conversion provides a new strategy to monitor the in vivo behavior of these MIONs.

### 3.3. Other Diagnosis Applications

Magnetic particle imaging (MPI) has the advantages of no tissue signal attenuation, a linear correlation between the signal and tracer concentration, and no ionizing radiation during detection. It has become an emerging tomographic imaging technology that is expected to enter clinical applications, especially in lung and other organs that are difficult to be imaged by MRI [82]. The earliest tracer used in MPI technology is SPIONs, and so far it is still the dominant tracer materials [82]. The Ms of the MPI tracer is decisive for its imaging performance. The Ms of Fe@Fe_3_O_4_ NPs are reported to be as high as 176 emu/g, which enables good MPI performance [83]. Changes in the crystallinity of MIONs can also alter the Ms, thereby affecting their MPI performance [84]. Magnetomotive optical coherence tomography (MMOCT) is another type of imaging technology based on MIONs, and the image contrast is derived from dynamic magnetomotive force. Unlike MRI and MPI, MMOCT requires a magnetic field as low as 0.08 T [85] and can detect ultra–low concentrations of tracers. MMOCT has been shown to be able to image tumor models in animals [12]. Due to its ability to detect the movement state of MIONs particles, it has also recently been used for real–time monitoring of magnetic hyperthermia [86].

### 3.4. Magnetic Hyperthermia

Hyperthermia is a treatment with a long history. MIONs have the ability to convert the energy of an alternating magnetic field (AMF) into heat. Hyperthermia using this magnetothermal effect is called magnetic hyperthermia (MHT). As a means of in situ hyperthermia, magnetic hyperthermia can kill tumor tissues more accurately, and it is not limited by the depth of tissue penetration. It has developed many application scenarios in the field of tumor therapy [87,88,89]. The improvement of the efficiency of tumor magnetic hyperthermia depends on the improvement of specific absorption rate (SAR), and SAR is directly proportional to the Ms of MIONs [90]. It has been proven that the Ms of MIONs are strongly related to their size [39]. SPIONs with a diameter of less than 20 nm have a SAR in the range of hundreds of W/g [42]. In theory, the SAR of MIONs could be improved by increasing the size of MIONs. Paradoxically, larger–sized MIONs also exhibit ferromagnetic/ferrimagnetic properties. The existence of remanence disfavors colloidal stability of the MIONs, which in turn can decrease its SAR. In recent years, some MIONs with special magnetic domain structures have gradually shown their advantages. In one example, iron oxide nanorings of a specific size will exhibit vortex magnetic domains. The magnetic domains of this structure are closed loops connected end to end. While maintaining high ferrous hysteresis loss, the residual magnetization is kept at zero, achieving a SAR exceeding 2000 W/g while having excellent colloidal dispersion [42]. The FePt@IONP synthesized by Yang et al. [91] also showed magnetic domains in the vortex state. This structure reduced the magnetic dipole–dipole interaction between FePt@IONP nanoparticles, prevented them from gathering in the remaining state, and improved the colloidal stability. Exchange coupling between FePt core and iron oxide shells can enhance the magnetic anisotropy of FePt@IONP, thereby improving its SAR and enabling more efficient magnetic hyperthermia. In addition, during the MRI scan of this FePt@IONP, the presence of a high main magnetic field induced the formation of NP chains and introduces an increase in the local uneven dipole field that ultimately enhances the T2 relaxation performance. Zhang et al. [92] grew a multi–domain eMION through the biomineralization process of capsules. The encapsulin–produced magnetic IONs (eMIONs) consist of FeO subdomains containing Fe_3_O_4_ with ~100% crystallinity. The SAR of eMIONs can reach 2390 W/g, and their nanozyme activity was also enhanced under the action of AMF, showing excellent tumor therapy capabilities, and the entire therapy process could be monitored in real time using MRI.

### 3.5. Chemodynamic Therapy

In an acidic environment, Fe^2+^ in MIONs can catalyze the Fenton reaction:

Fe^3+^ + H_2_O_2_ = Fe^2+^ + HO_2_• + H^+^

Fe^2+^ + H_2_O_2_ = Fe^3+^ + •OH + OH^−^

**Figure 3:**
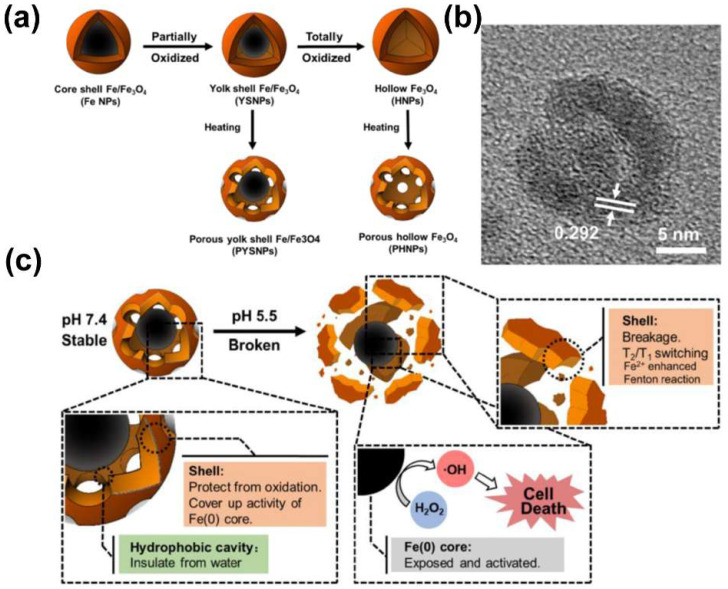
(**a**) Schematic illustration of PYSNP preparation in [64]. (**b**) HRTEM images of PYSNP. (**c**) Schematic illustration of the pH activated Fe release in PYSNPs. Reprint with permission from [81]. Copyright 2020 Elsevier Ltd., Amsterdam, The Netherlands.

Due to its similar behavior to peroxidases such as horseradish peroxidase (HRP), the ability of MIONs to catalyze the Fenton reaction is also called peroxidase–like activity of a nanozyme. This reaction can generate a large amount of ROS in the tumor cells, break the redox balance of tumor cells, and then damage tumor cells [93]. This phenomenon is known as chemodynamic therapy (CDT). However, since the pH in the tumor microenvironment does not meet the optimal conditions for the Fenton reaction [94,95], the direct application of MIONs in tumor CDT is limited. Hence, strategies to improve the Fenton reaction activity of MIONs are vital for their CDT efficacy. Liang et al. [81] synthesized a porous yolk–shell Fe/Fe_3_O_4_ nanoparticles (PYSNP, Figure 3a,b), which used Fe_3_O_4_ shell to protect Fe^0^ from oxidation and to deliver it to the tumor microenvironment. Disintegration of the nanoparticles into fragments results in the transformation of its MRI imaging performance from T2 to T1, realizing image tracking of the delivery process. Finally, the relatively higher Fenton reactivity of the exposed Fe^0^ was used to achieve high–efficiency tumor CDT (Figure 3c). Aiming at the problem that the concentration of H_2_O_2_ in cancer cells is not sufficient for effective CDT [96], Du et al. [97] used mesoporous silica nanoshell to connect ultrasmall SPIONs with Au nanorods and constructed a core–shell–satellite nanomaces (Au @ MSN@IONP), AuNR can convert near infrared light into heat to cause heat stress in cancer cells and to generate a large amount of H_2_O_2_. The H_2_O_2_ then acted as a substrate of MIONs for Fenton reaction, achieving a highly specific anticancer effect, and inhibited PI3K/Akt/FoxO axis which is closely related to the redox regulation and survival of breast cancer cells. MIONs–enhanced MRI provided imaging guidance for the photodynamic therapy. Low–intensity focused ultrasound (LIFU) has also recently been used to enhance CDT by Deng and coworkers [98]. They loaded vitamin C (Vc) and SPIO together inside the PLGA nanospheres to fabricate PLGA–SPIO&Vc. The PLGA–SPIO&Vc were delivered to tumor cells through magnetic targeting and EPR effect. Subsequently, the cavitation and oscillation effects of LIFU were used to promote the release of Vc and to lower the environmental pH. Vc also worked as a H_2_O_2_ precursor to provide the materials for Fenton reaction. Photoacoustic imaging was used by them to detect the progress of the Fenton reaction.

Interestingly, the magnetothermal effect of MIONs can also be used to enhance its intrinsic catalytic activity. Exposing MIONs to AMF was shown to effectively enhance their peroxidase nanozyme activity without changing the bulk temperature of the solution, and the degree of rate enhancement has a linear dependence on the SAR of MIONs [99]. Zhang et al. studied the influence of the magnetothermal effect of MIONs on the activity of the natural enzyme glucose oxidase (GOx) [59]. They found that the degree to which GOx is enhanced by AMF stimulation is related to the distance between the MIONs and GOx. With an optimal distance of 1 nm, the hybrid MIONs–GOx catalyst shows the highest cascade activity to produce a large amount of ROS and achieves the best tumor inhibition effect in a mouse breast cancer model.

## 4. Implementation of Magnetotheranostic Based on Magnetic Nanoplatforms

MIONs can be coupled with a variety of nanocomposites, so that multiple diagnosis and therapy technologies can be integrated on a single nanoplatform. This includes magnetotheranostics based on magnetic field, magnetoptical theranostics, PET or CT, fluorescence imaging, drug delivery, etc. MIONs can be used as a nanoplatform to integrate almost all existing diagnosis and therapy methods. The following will describe functional modifications of MIONs for integration of magnetic diagnosis and treatment.

### 4.1. Magnetotheranostics Based on Magnetic Nanoplatforms Only

As mentioned in Section 3, MIONs have their own therapeutic and diagnostic functions such as MRI, MPI, MHT, and CDT. Therefore, improvements on MIONs are beneficial for their magnetotheranostics performances. The Ms of MIONs received great attention from researchers at an earlier time [34], because the improvement of Ms will simultaneously enhance the T2–weighted imaging performance of MIONs and the thermal conversion efficiency of MHT. Recent work in this area has become more diversified, and one direction is T1–T2 dual–modality imaging combined with treatment. Liu et al. [90] synthesized wüstite Fe_0.6_Mn_0.4_O nanoflowers. Unlike the antiferromagnetic bulk wüstite, Fe_0.6_Mn_0.4_O nanoflowers exhibit ferromagnetism, which may be due to exchange coupling effect. The as–prepared nanoflowers exhibit excellent magnetic induction heating effects (SAR can reach 535 W/g), which could induce tumor regression in breast cancer through MHT. The longitudinal relaxation rate r1 and lateral relaxation rate r2 of Fe_0.6_Mn_0.4_O nanoflowers are as high as 4.9 and 61.2 mM^−1^ _[Fe]+[Mn]_·s^−1^, respectively. These nanoflowers showed both T1 and T2 enhancing properties in the mouse glioma model. Different from this static T1–T2 dual–modal contrast agent, another type is dynamic T1–T2 dual–modal contrast agents. They can present two states of T1 or T2 contrast, and certain events will prompt the transition between the two states. This is generally accomplished by disintegrating large particles into small particles (T2 to T1) or aggregation of small particles into large particles (T1 to T2). An example of the conversion of T2 enhancement to T1 enhancement is listed in Section 3.2 [81]. In the work by Zhou et al. [100], the ultrasmall SPIONs aggregated into clusters in the tumor in situ, resulting in the conversion of T1 enhancement to T2 enhancement. They used hyaluronic acid (HA) to encapsulate ultrasmall SPIONs, which showed T1 enhanced performance before penetrating into the tumor. After entering the tumor area, the surface–modified HA was degraded by the abundant hyaluronidase, which decreased the colloidal stability of ultrasmall SPIONs and caused aggregation of the nanoparticles into clusters, resulting in enhanced T2 imaging performance and weakened T1 imaging performance. Although the therapy performance of the designed ultrasmall SPIONs was not investigated in this work, its penetration–aggregation design still provided a strategy for future magnetotheranostics.

**Figure 4:**
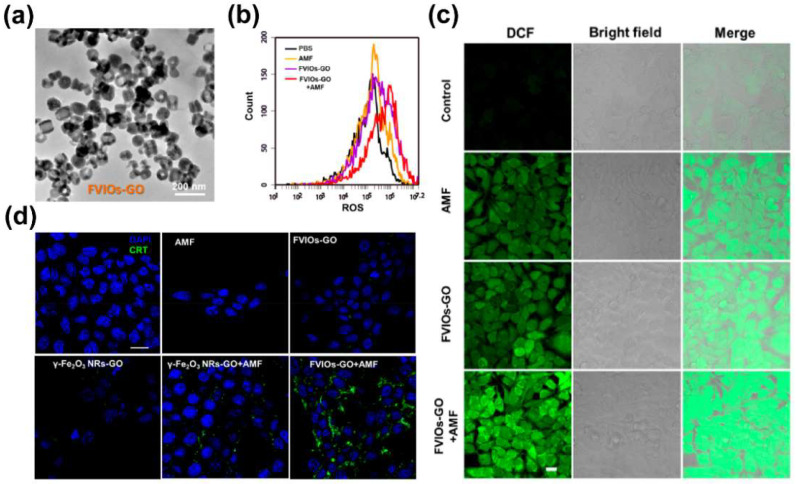
(**a**) TEM image of FVIOs–GO. (**b**) ROS generation of 4T1 breast cancer cells after treatment in a flow cytometry assay. (**c**) Confocal laser scanning microscope (CLSM) images of 4T1 cells treated with complete media (control), AMF, FVIOs–GO only, and FVIOs–GO–containing media under AMF. Generation of ROS was monitored using DCFH–DA (green). Scale bars for all images are 20 μm. (**d**) Confocal images showing the CRT exposure on 4T1 tumor cells in vitro after treatment with γ–Fe_2_O_3_ nanorings+AMF or FVIOs–GO–mediated magnetothermodynamic (MTD) therapy. Scale bars for all images are 20 μm. Reprint with permission from [57]. Copyright 2020 American Chemical Society.

Another direction that has received widespread attention is the tumor immune effect caused by ROS produced in CDT. The FDA–approved iron supplement ferumoxytol (FMX) was confirmed to be able to polarize tumor–associated macrophages from the anti–inflammatory M2 phenotype to the pro–inflammatory M1 phenotype through the induced ROS [101]. Further studies have shown that the ROS generated by iron oxide–loaded nanovaccines (IONVs) can promote the presentation of tumor antigens, mediate tumor immune cell infiltration, and stimulate non–toxic long–term protective antitumor immunity [102]. MRI can provide real–time location of IONVs in this work. Liu et al. [57] reported that the Fenton reaction activity of MIONs (FVIOs–GO, Figure 4a) could be enhanced under the action of AMF, causing the massive production of ROS (Figure 4b,c), which in turn lead to calreticulin (CRT) of cancer cells migration from the endoplasmic reticulum to the outside of the plasma membrane (Figure 4d), causing immunogenic cell death (ICD). MRI could be also used to monitor the location of MIONs.

### 4.2. Integration of Magnetic Nanoplatforms with Phototheransotics

**Figure 5:**
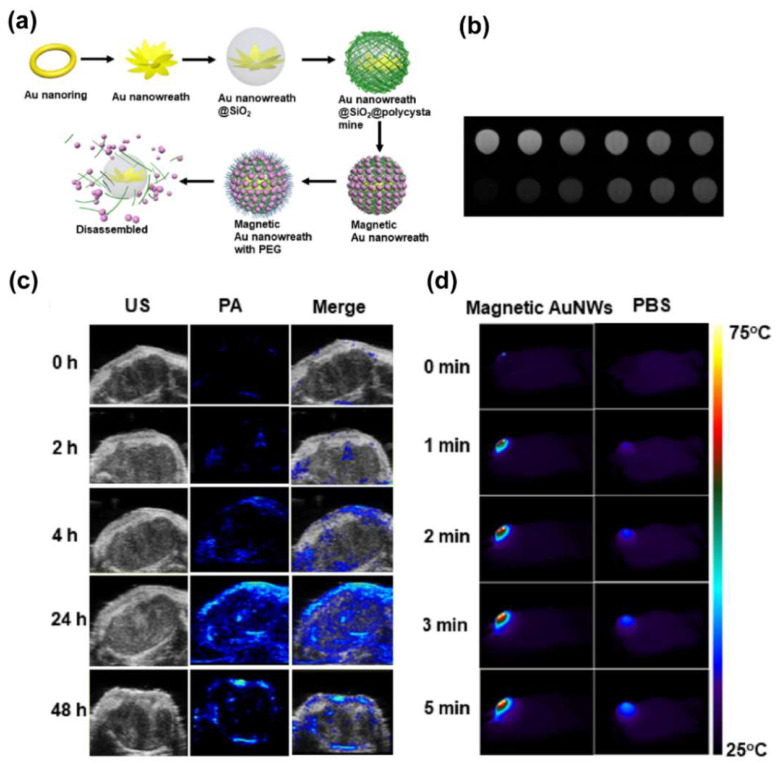
(**a**) Schematic illustration of the synthesis of magnetic gold nanowreath. (**b**) Corresponding T1–weighted images (top, disassembled; bottom, assembled) of magnetic AuNWs. Concentrations of Fe are 0.5, 0.25, 0.125, 0.0625, 0.03125, and 0.01563 mM (from left to right). (**c**) Ultrasonic (US), photoacoustic imaging (PA), and merged images of tumor before injection (0 h) and at 2, 4, 24, and 48 h after intravenous injection of magnetic AuNWs upon irradiation by an 808 nm pulsed laser. (**d**) Representative thermal images of U87MG tumor bearing mice after injection of magnetic AuNWs and PBS. The tumors were irradiated by an 808 nm CW laser at 0.75 W/cm^2^. Reprint with permission from [104]. Copyright 2018 American Chemical Society.

Similar to magnetotheranostics, comprehensive application of the optical properties of nanomaterials in therapeutic diagnostics are called phototheranostics, covering technologies such as photothermal therapy (PTT), photodynamic therapy (PDT), and photoacoustic imaging (PAI). Corresponding to the magnetic core in magnetotheranostics, the phototheranostics nanoplatform needs a photosensitizer as its core. Among the various photosensitizers, noble metal nanoparticles, especially Au nanoparticles [103] can efficiently complete energy conversion through the localized surface plasmon resonance (LSPR) effect, and are often used to form a hybrid magnetoptical theranostics nanoplatform with MIONs for therapeutic diagnostics. Liu et al. [104] assembled SiO_2_–coated Au nanowreaths (AuNWs) with ultrasmall SPIONs through molecules containing polycystamine blocks (Figure 5a). After sensing the GSH in the tumor cells, the disulfide bond of polycystamine was cleaved, causing the disassembly of ultrasmall SPIONs from the surface of AuNWs, and the MRI contrast performance of the ultrasmall SPIONs changed from T2 enhancement to T1 enhancement (Figure 5b). The released AuNWs were used for photothermal therapy and photoacoustic imaging (Figure 5c,d). Amphiphilic Janus nanoparticles with hydrophilic PEG–modified AuNPs and hydrophobic poly(lipid hydro–peroxide)–co–poly(4–vinylpyrene) (PLHPVP) modified MIONs were reported to form a double–layered vesicle [17]. Its MRI and PAI performance could be enhanced through magnetic dipole interaction and strong plasma coupling. The inner cavity of this vesicle could be loaded with DOX to deliver the drug into tumor cells, and the outer side was modified with the radioactive isotope ^64^Cu for PET imaging. After entering the tumor cells, the acidic environment disassembled the vesicles, and PLHPVP became hydrophilic under the influence of H^+^ and allowed Fe^2+^ to contact the environment. Fe^2+^ further reacted with LHP to generate ROS and cooperates with DOX to kill tumor cells. Metal sulfide is also one of the common photosensitizers. IONPs anchored on titanium disulfide (TiS_2_) nanosheets have strong absorption and excellent magnetic properties in the second near–infrared (NIR–II) window and had been developed as NIR–II PAI and MRI–guided photothermal therapy, combined with immunotherapy to prevent tumor recurrence [105]. Besides MIONs, iron(II) sulfide nanoparticles could also exhibit superparamagnetism, and their strong absorption in the near–infrared region enables them to be used as PTT agents. In addition, its ultra–high r2 relaxivity makes it an excellent T2 contrast agent [106]. In addition to these inorganic materials, some organic materials having light–to–heat conversion capabilities are also used in magnetotheranostics. Polydopamine (PDA) has traditionally been used to modify MIONs [21] for better colloidal stability and biocompatibility. PDA can also be used as a photothermal agent to mediate photothermal therapy. T2–weighted MRI was used for image–guided therapy. Porphyrin is a bioinspired organic photosensitizer. The porphyrin derivative meso–tetrakis(4–carboxyphenyl)porphyrin (TCPP) could be excited by the Cerenkov luminescence of ^89^Zr connected to the MIONs platform to generate ROS, which was used for PDT without external light source [107]. In this example, MIONs were designed as Zn_0.4_Mn_0.6_Fe_2_O_4_ to obtain optimized Ms for magnetic targeting. In another study, protoporphyrin IX (PpIX) was used to coat SPIONs to form clusters, which could realize the integration of diagnosis and therapy of MRI and PDT [108].

### 4.3. Integration of Magnetic Nanoplatforms with Fluorescence Imaging

Fluorescence imaging (FI) relies heavily on the penetration of light in tissue, which limits its application in the field of in vivo diagnosis and therapy. However, because fluorescent molecules can be designed to achieve highly specific responsiveness, fluorescence imaging is often used as an auxiliary imaging method for the magnetotheranostics nanoplatform to monitor its response behavior in the body. Zhou et al. [109] designed a nanoplatform capable of detecting tumor hypoxic environment, composed of ultrasmall SPIONs and assembly–responsive fluorescent dyes (NBD), and used nitroimidazole derivatives as hypoxia–sensitive detectors. Zhou et al. [97] designed a nano–platform capable of detecting the hypoxic environment of tumors, consisting of USION and assembly–responsive fluorescent dyes (NBD), and used nitroimidazole derivatives as hypoxia–sensitive detectors. In an oxygen–rich environment, ultrasmall SPIONs showed enhanced T1 performance. Under hypoxic conditions, NBD cross–linked irreversibly, leading to self–assembly of ultrasmall SPIONs, and thus its contrast performance changed from T1 MRI to T2. At the same time, the cross–linking of NBD increased its fluorescence intensity, indicating that the oxygen environment level in this area had decreased. In another example, combination of MRI and fluorescence imaging was used to monitor the progress of CDT [110]. NQ–Cy, MIONs, and GOx were mixed and loaded in the micelles of DSPE–PEG–FA. The MRI capability of MIONs provided information to monitor the delivery of the micelles. Next, the increase of fluorescence at 830 nm indicated successful release of NQ–Cy into the cytoplasm. Subsequently, the released MIONs and GOx produced a large amount of ROS in the cytoplasm, causing cell oxidative stress and an increase in NQO1 enzyme expression. Then NQ–Cy was decomposed by NQO1, and its emission wavelength shifted from 830 to 670 nm, indicating that CDT had effectively activated the oxidative stress of cells. 

### 4.4. Integration of Magnetic Nanoplatforms with CT&PET/SPECT

**Figure 6:**
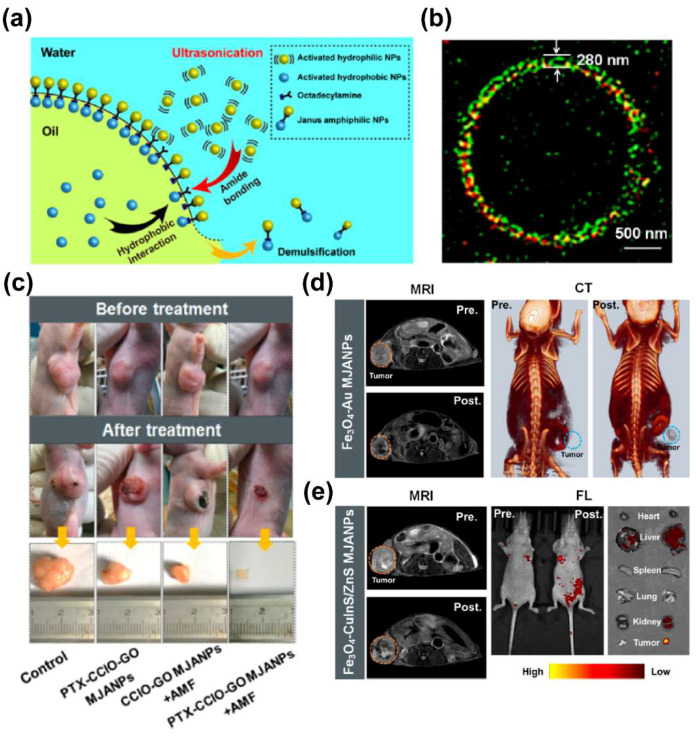
(**a**) Schematics of the ultrasonatically triggered interfacial assembly process. (**b**) SIM image of the Pickering emulsion. Fluoresceinamine was used to label graphene oxide nanosheets (green), and rhodamine B isothiocyanate (RITC) was used to label Co cluster–embedded IONPs (red). (**c**) Photographs of pre– and post–MHT treatment mice. (**d**) In vivo T2–weighted MRI and CT images of tumor–bearing mice pre– and post–injection of Au NPs−MIONs MJANPs. Arrowheads indicate the tumor. (**e**) In vivo T2–weighted MRI and fluorescence images of tumor–bearing nude mice pre– and postinjection of CuInS/ZnS−MIONs MJANPs. Arrowheads indicate the tumor. Reprint with permission from [16]. Copyright 2019 American Chemical Society.

Although iodine–based contrast agents for CT have been well developed, certain components of the magnetotheranostics nanoplatforms also have the ability to act as CT contrast agents. The use of CT to guide the diagnosis and therapy of the magnetic nanoplatforms has remarkable application prospects. A relatively common example is the gold–magnetic composites, wherein the high CT value of gold enables it to be traced by CT [111]. Liu et al. developed an ultrasonication–triggered interfacial assembly approach (Figure 6a,b) to synthesize magnetic Janus amphiphilic nanoparticles (MJANPs) for image–guided cancer MHT (Figure 6c) [16]. Au NPs–MIONs MJANPs made of Au NPs and MIONs could achieve MRI/CT dual–modality imaging and could be used to guide MHT (Figure 6d). Similarly, if CuInS/ZnS NPs and MIONs were used to make CuInS/ZnS NPs–MIONs MJANPs, MRI/FI dual–modality imaging can be used to guide MHT (Figure 6e). In the bismuth ferrite (BFO) nanoplatform designed by Feng et al., BFO nanoparticles could achieve CT contrast effects similar to iohexol [112]. The 2D–Ta_3_C_4_–MIONs designed for PTT and T2–weighted MRI also have a CT value exceeds that of the clinically used CT contrast agent iopromide [113]. PET/SPECT imaging relies on the labeling of radioisotopes. Combining radioisotopes with the magnetotheranostic nanoplatforms is a common method for multimodal imaging. These radioisotopes include ^18^F, ^59^Fe, ^64^Cu, ^68^Ga, ^89^Zr, ^99m^Tc [114,115,116,117]. In a comparative study by Zhang et al. [114], two modification strategies using radioisotopes (^59^Fe) in the core of MIONs and radioisotopes (^64^Cu) labeling in the shell were systematically compared. They believed that the shell labeling was relatively more attractive due to its flexible design, easy operation, and low radiation risk, but the core labeling had better stability for in vitro tests.

### 4.5. Magnetic Nanoplatforms Carrier Based Drug Delivery

**Figure 7:**
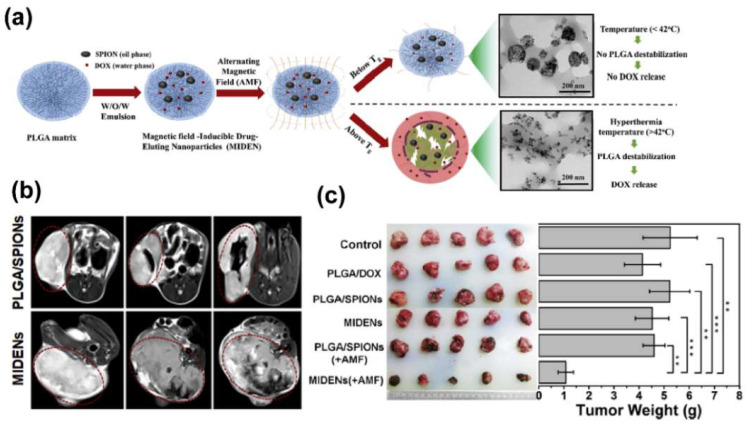
(**a**) Schematic illustration showing the formation of magnetic field inducible drug–eluting nanoparticles (MIDENs). (**b**) T2 MRI of CT26 xenografted mice administered intratumorally with PLGA/SPIONs and MIDENs (10 mg_[Fe]_/kg body weight). Tumor regions are marked with a red circle. (**c**) Photographs of tumor tissues and weights of tumors collected 15 days post treatment (*n* = 5). The statistical significance was evaluated using Student’s *t*–test. ** *p* < 0.01, *** *p* < 0.001. Reprint with permission from [126]. Copyright 2018 Elsevier Ltd., Amsterdam, The Netherlands.

Chemotherapy is one of the main methods in current tumor therapy. There are currently about 80 chemotherapeutic drugs in clinical service, but these drugs can inevitably cause damage to normal tissues at therapeutic doses [118]. In order to improve the chemotherapy efficiency of drugs on tumors and to reduce their toxicity to normal tissues, it is necessary to develop an efficient drug delivery system. The magnetotheranostic nanoplatform has become a representative delivery system due to its adjustable size, easy image tracking, and clear metabolic pathway. The delivery mechanism of MIONs can be divided into passive targeting and active targeting. Passive targeting mainly depends on the enhanced permeability and retention (EPR) effect of MIONs. Although the mechanism still needs to be further investigated [119,120], the EPR effect can indeed enhance the enrichment of nanoparticles (not just MIONs) in the tumor area [5]. In addition, the ligands of tumor characteristic markers can be employed to functionalize MIONs in order to give them the ability to actively target tumors [6], and for more efficient drug delivery. In a recent work, paclitaxel (PTX) and cisplatin (CDDP) have been loaded into the carboxymethyl dextran coating of the clinical iron supplement FMX, and actively target gliomas through HMC, which was an organic anion transport polypeptide targeting agent with near–infrared fluorescence. This system was used for MRI/FI visualized drug delivery of glioblastoma multiforme (GBM) [121]. Liu et al. [122] designed a delivery system with a Yolk–shell structure. The vesicles were composed of PEG–PPS–SS–PEG and loaded with ultrasmall SPIONs and DOX, which were encapsulated together by a polyacrylic acid coating. In tumor cells, vesicles were disintegrated under the influence of GSH. The complex of ultrasmall SPIONs and DOX was then separated, so that the drug release process could be monitored by an enhancement in T1 MRI. The complex microenvironment of the tumor tissue could limit the penetration of the nanoplatforms [123,124]. In the work of Zhang et al., hyaluronidase was used to reduce the viscosity of the tumor ECM and to improve the tumor penetration of the magnetotheranostic nanoplatform [125]. In this platform, ultrasmall SPIONs were stabilized with layered double hydroxide (LDH), hyaluronic acid (HA) was modified on the outside of LDH and DOX was loaded inside the LDH. LDH–Fe_3_O_4_–HA/DOX could efficiently penetrate into the tumor pretreated with hyaluronidase and enter the lysosome in the tumor cell through the HA–CD44 pathway. The LDH sensed the pH decrease in the lysosome and released DOX to kill tumor cells. The entire delivery process was monitored by T1–weighted MRI. On the other hand, the magnetothermal effect of MIONs was designed to remotely control drug release [126]. Temperature–sensitive poly(lactic–co–glycolic acid) (PLGA) was used to coat SPIONs and DOX (Figure 7a). With the help of T2–weighted MRI (Figure 7b) to monitor the enrichment of the nanoplatforms in the tumor area, AMF was then applied to exert magnetothermal effect to the system. When the temperature rose above 42 °C, PLGA underwent a phase change, releasing DOX for chemotherapy (Figure 7c). Liu et al. combined tumor penetration and drug controlled release into one magnetotheranostics platform [127]. They modified temperature–sensitive hyperbranched PEI on ferrimagnetic vortex–domain iron oxide nanorings (FVIOs) with DOX loaded inside the PEI. The 0.1 kHz low–frequency magnetic field induced the magnetic force effect of FVIOs to effectively penetrate the magnetotheranostics nanoplatform deep into the tumor tissue. The cells then took up the positively charged magnetotheranostics platform. After entering the cells, the 360 kHz intermediate frequency magnetic field was turned on to raise the surface temperature of FVIOs, causing phase transition of the temperature–sensitive PEI. PEI shrank violently and then DOX was released. The sudden increase in intracellular DOX concentration during this release process was sufficient to effectively kill DOX–resistant MCF–7 breast cancer cells. Some smart micro–nano platforms with more complex structures are often called “micro/nano–robots” because their behaviors can be precisely manipulated under the external magnetic fields. Park et al. developed a degradable hyperthermia micro–robot (DHM) with a three–dimensional spiral structure [7], which contains MIONs and 5–fluorouracil (5–FU). The movement of DHM was controlled by a rotating magnetic field (RMF), and the AMF was then applied for magnetic hyperthermia. Upon AMF stimulation, 5–FU can be released in different modes for precise chemotherapy. MIONs can carry multiple types of drugs for collaborative treatment. In the work by Li et al. [128], PEI–PEG–coated MIONs were used to load gemcitabine and miRNA for pancreatic cancer treatment. They further installed the CD44v6 targeting molecule on the magnetic nanoplatform to improve the delivery efficiency, while the delivery process can be monitored using MRI.

## 5. Summary and Perspectives

In summary, we focused on reviewing the design of the magnetic nanoplatforms in the integration of tumor diagnosis and therapy in recent years. In the past, magnetotheranostics has mostly referred to the combination of MRI and other therapy technologies [95], which is a mixture of the MRI–enhancing properties of its magnetic core, MIONs, and materials with other properties, such as gold magnetic materials. With the in–depth study of the MIONs by researchers, the therapeutic function of MIONs (such as MHT and CDT) has gradually been integrated to realize magnetotheranostics. Some new imaging methods based on MIONs, such as MPI, MMOCT, and even magnetoacoustics ultrasound imaging [129], are also emerging and are expected to enrich future magnetotheranostics nanoplatforms.

MIONs as MRI contrast agents, magnetothermal agents, and iron supplements have been approved for clinical use. Although the magnetotheranostics nanoplatforms based on MIONs have potential for clinical implementation, there are still several issues to be solved. First of all, the current preparation cost of the magnetotheranostics nanoplatforms is relatively high, the stability of large–scale synthesis is questionable, and the quality control lacks evaluation standards. Secondly, the EPR effect of nanoparticles has not been effectively proved. Finally, in some complex magnetotheranostics nanoplatform systems, the potential toxicity of each component has not yet been resolved. As more researchers focus on elucidating these matters, we foresee that magnetotheranostics nanoplatforms will serve as an important new theranostics technology in future clinical practice.

## Data Availability

Data are contained within the article.
